# Whisking Kinematics Enables Object Localization in Head-Centered Coordinates Based on Tactile Information from a Single Vibrissa

**DOI:** 10.3389/fnbeh.2016.00145

**Published:** 2016-07-19

**Authors:** Anne E. T. Yang, Mitra J. Z. Hartmann

**Affiliations:** ^1^Department of Mechanical Engineering, Northwestern University, EvanstonIL, USA; ^2^Department of Biomedical Engineering, Northwestern University, EvanstonIL, USA

**Keywords:** whisker, biomechanics, touch, orienting, trigeminal, superior colliculus

## Abstract

During active tactile exploration with their whiskers (vibrissae), rodents can rapidly orient to an object even though there are very few proprioceptors in the whisker muscles. Thus a long-standing question in the study of the vibrissal system is how the rat can localize an object in head-centered coordinates without muscle-based proprioception. We used a three-dimensional model of whisker bending to simulate whisking motions against a peg to investigate the possibility that the 3D mechanics of contact from a single whisker are sufficient for localization in head-centered coordinates. Results show that for nearly all whiskers in the array, purely tactile signals at the whisker base – as would be measured by mechanoreceptors, in whisker-centered coordinates – could be used to determine the location of a vertical peg in head-centered coordinates. Both the “roll” and the “elevation” components of whisking kinematics contribute to the uniqueness and resolution of the localization. These results offer an explanation for a behavioral study showing that rats can more accurately determine the horizontal angle of an object if one column, rather than one row, of whiskers is spared.

## Introduction

Many rodents, including rats, exhibit 5 – 12 Hz “whisking” motions of the large facial vibrissa as they tactually explore the environment. During tactile exploration with whiskers, rats can orient quickly and precisely to an object even though there are very few proprioceptors in the whisker muscles (Ebara et al., 2002; Moore et al., 2015). A large open question, then, is how the rat might localize an object in head-centered coordinates without muscle-based proprioception. Specifically, how does the rat know the angular position of the whisker (and hence the object) at the instant of whisker-object contact? Several possible answers to this question have been proposed by three behavioral studies that have quantified the degree to which rodents can localize an object in the horizontal plane (Knutsen et al., 2006; Mehta et al., 2007; O’Connor et al., 2010).

Mehta et al. (2007) propose that the rat integrates a binary touch signal with kinesthetic information about the angular position of the vibrissa at the time of contact. The authors suggest that the kinesthetic signal could be generated either in the periphery, via “whisking” neurons of the trigeminal ganglion (Zucker and Welker, 1969; Szwed et al., 2003, 2006; Leiser and Moxon, 2007; Khatri et al., 2009; Wallach et al., 2016) or from a cortical reafferent signal (Mehta et al., 2007; Curtis and Kleinfeld, 2009; Hill et al., 2011; Moore et al., 2015). Similarly, Knutsen et al. (2006), suggest that temporal coding (e.g., the interval elapsed between whisking onset and object contact) could provide information about the angular location of the object in head-centered coordinates (Szwed et al., 2003, 2006; Knutsen and Ahissar, 2009; Wallach et al., 2016). O’Connor et al. (2010) who worked in mice, indicate that differences in the bending of the whisker are observed for different angular locations, but experimental resolution of the study did not permit detailed quantification of these effects.

An intriguing alternative hypothesis was proposed in a fourth study that examined three dimensional (3D) whisking kinematics (Knutsen et al., 2008). In this study, the dorsal-ventral elevation as well as the whisker’s roll about its own axis were found to be coupled to the protraction angle. In the discussion, the authors suggest that the roll of the whisker could provide a mechanism for the rat to determine where in the whisking cycle an object has been contacted.

Our laboratory recently developed a 3D quasi-static model of whisker bending that allows us to test this hypothesis in simulation. The model computes all six components of force ($\overset{⇀}{F}$) and moment ($\overset{⇀}{M}$) at the vibrissal base as a rat whisks against a peg (Huet et al., 2015; Huet and Hartmann, 2016). We can therefore determine to what extent the information present in these mechanical signals sufficient to yield the location of the object.

In behavioral terms, the present work asks: are the tactile signals obtained by a single whisker and transmitted to the follicle – in *whisker* centered coordinates – sufficient to uniquely determine the location of an object so that the animal can orient to it in *head*-centered coordinates?

## Materials and Methods

### Position and Orientation of the Head and the Pegs Surrounding the Head

The goal of the present work was to simulate the 3D tactile-mechanical information available to the animal under conditions that resembled as closely as possible those of the relevant behavioral experiments (Knutsen et al., 2006; Mehta et al., 2007; O’Connor et al., 2010). All of these studies either worked in the head-fixed animal or indicated that head motions were very small. Given that the kinematic equations for 3D whisker motion were also obtained from the head-fixed animal (Knutsen et al., 2008), the present work simulates whisking in the head-fixed rat. Therefore, in simulation, an anatomically accurate model of the rat head and vibrissal array (Towal et al., 2011) was placed with the snout at the origin and with bregma aligned with lambda in the horizontal plane.

**FIGURE 1:**
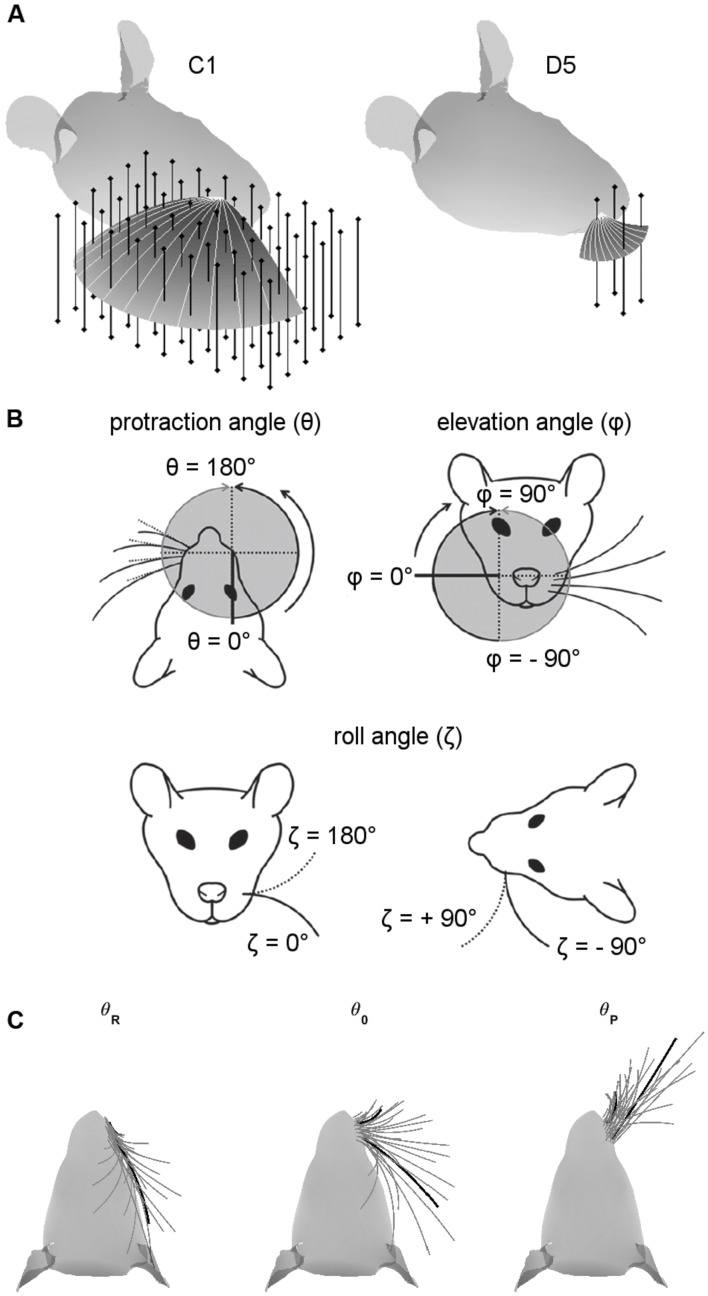
**Coordinate systems, variables, and parameters used in the simulations.** **(A)** Examples of peg locations relative to the head. The two figurines illustrate the 3D protraction of the C1 and D5 whiskers. The head is oriented so that bregma is aligned with lambda in the horizontal plane. Protraction of the whisker is shown as a curved surface, with shading proportional to the *z*-coordinate (height). Solid light gray lines within the surface show the whisker shape in 10° increments during the protraction. Vertical bars represent pegs evenly distributed on an *x-y* grid. Pegs are illustrated at reduced resolution as 4-mm apart in both x- and y- directions for visual clarity, but all simulation results were obtained with 1-mm spacing. **(B)** Angle definitions. The angle 𝜃 is the protraction angle, 𝜃 is the elevation angle, and ζ is the roll of the vibrissa about its own axis. Figure adapted from (Hobbs et al., 2016a). **(C)** Range of whisking. To ensure coverage of the full whisking range, all simulations started with the whisker retracted to be tangent to the rat’s face in the top–down view. Each whisker was then protracted by 120° or until the tip of the whisker the *y*-coordinate of the whisker-tip started to decrease. The C1 and D5 vibrissae are drawn as thick black lines for visual reference.

Vertical pegs (infinitely tall) were placed on a grid in Cartesian coordinates surrounding the head (**Figure 1A**). Pegs were spaced 1 mm apart. The grid of pegs occupied the complete region of space that could be reached by the whiskers during retraction and protraction over the range defined in Section “Choice of Protraction Amplitude”. Simulations were run independently for each whisker. All 31 whiskers in the array were simulated. Some whiskers in the E-row had a strong concave-downward orientation, and this orientation was nearly parallel to the peg for some peg locations. In these cases the mechanics were not well defined, and these peg locations were eliminated in post-processing.

Simulations included only those pegs located between 30 and 90% of the whisker arc length in order to avoid extremely large mechanical signals near the base and immediate slip-off from the peg near the tip of the whisker.

### Simulating Whisking Kinematics

Simulations employed the standard variables illustrated in **Figure 1B**, where 𝜃 represents the horizontal (protraction) angle of the whisker, φ is the elevation angle, and ζ is the roll of the whisker about its own axis, as defined by the whisker’s intrinsic curvature (Knutsen et al., 2008; Towal et al., 2011).

As in several previous studies (Huet and Hartmann, 2014, 2016; Hobbs et al., 2015, 2016a,b), the horizontal resting angle (𝜃_0_) and the resting orientation of each whisker about its own axis (ζ_0_) were obtained from Towal et al. (2011). The elevation resting angles (φ_0_) were obtained from Knutsen et al. (2008). Note that we obtained 𝜃_0_ and ζ_0_ from Towal et al. (2011) because these angles are not provided in Knutsen et al. (2008), but φ_0_ was obtained from Knutsen et al. (2008) because the parametrization in Towal et al. (2011) yields unrealistically low values of φ_0_ for the A-row whiskers (Huet and Hartmann, 2014).

All simulations were initially run using equations for whisking kinematics obtained from awake behaving animals by Knutsen et al. (2008). These equations were obtained in a coordinate system in which the horizontal plane was defined by the line connecting the nose and the anterior-most corner of the rat eye.

We realized, however, that more accurate results would be obtained if the kinematic equations of Knutsen et al. (2008) were converted to the same coordinate system as the morphological model of the rat, in which the horizontal plane is defined by bilateral alignment of the whisker rows (Towal et al., 2011). We therefore performed this coordinate-system conversion. The original and converted kinematic equations are shown in the top and bottom halves of **Table 1.** All simulations were then completely rerun using the new, converted equations, and results compared with those obtained using the old equations.

**Table 1:** Equations used to simulate whisking kinematics.

Row	Equation for protraction	Equation for elevation	Equation for roll
**Kinematic equations in the coordinate system of Knutsen et al. (2008)**			
A	d𝜃 = 0.02°/timestep	φ = (56 ± 5.3) + 0.12d𝜃	ζ = ζ_0_ – (0.76 ± 0.08)d𝜃
B	d𝜃 = 0.02°/timestep	φ = (25 ± 9.4) + 0.30d𝜃	ζ = ζ_0_ – (0.25 ± 0.18)d𝜃
C	d𝜃 = 0.02°/timestep	φ = (-4.2 ± 6.3) + 0.30d𝜃	ζ = ζ_0_ + (0.22 ± 0.22)d𝜃
D	d𝜃 = 0.02°/timestep	φ = (-27.2 ± 7.7) + 0.14d𝜃	ζ = ζ_0_ + (0.42 ± 0.11)d𝜃
E	d𝜃 = 0.02°/timestep	φ = (-44 ± 7.6) + 0.02d𝜃	ζ = ζ_0_ + (0.73 ± 0.14)d𝜃
**Kinematic equations after converting to the coordinate system of Towal et al. (2011)**			
A	d𝜃 = 0.02°/timestep	φ = (53.3 ± 4.25) + (0.398 ± 0.005)d𝜃	ζ = ζ_0_ – (0.900 ± 0.026)d𝜃
B	d𝜃 = 0.02°/timestep	φ = (22.1 ± 4.69) + (0.591 ± 0.008)d𝜃	ζ = ζ_0_ – (0.284 ± 0.005)d𝜃
C	d𝜃 = 0.02°/timestep	φ = (-6.59 ± 5.30) + (0.578 ± 0.000)d𝜃	ζ = ζ_0_ + (0.243 ± 0.000)d𝜃
D	d𝜃 = 0.02°/timestep	φ = (-30.2 ± 5.21) + (0.393 ± 0.001)d𝜃	ζ = ζ_0_ + (0.449 ± 0.001)d𝜃
E	d𝜃 = 0.02°/timestep	φ = (-46.6 ± 4.64) + (0.217 ± 0.000)d𝜃	ζ = ζ_0_ + (0.744 ± 0.001)d𝜃


*Whisker angles φ and ζ are functions of the protraction angle 𝜃 and the row identity of the whisker. **(Top half)** Numerical constants in these equations were obtained from the experimental study of Knutsen et al. (2008). The incremental displacement d𝜃 is measured relative to the resting angle 𝜃_*0*_, which was obtained from Towal et al. (2011). The resting angle ζ_*0*_ is unique for each vibrissa and was obtained from Towal et al. (2011). Plus-minus values in equations for φ and ζ are error bounds from Knutsen et al. (2008). **(Bottom half)** The equations in the top half of the table were converted to the coordinate system of the morphological model. Details of the conversion are described in Section “*Materials and Methods*.”*

The conversion shown in **Table 1** was achieved in four steps.

First, we found the head pitch offset between the coordinate system of the morphological model and the coordinate system in which the kinematic equations were determined. The pitch difference was found to be a rotation of 13.8° clockwise, tipping the snout down. This pitch difference goes from the coordinate system of the morphological model to the coordinate system of the kinematic equations.

Second, recall that the resting elevation angle φ_0_ was originally obtained from the coordinate system of the kinematic equations. We therefore had to determine this angle in the coordinate system of the morphological model. To do this, all whiskers were first set to their resting azimuthal angles in the coordinate system of the morphological model (𝜃_0_). All whiskers were rotated by the 13.8° pitch offset to be in the same coordinate system as the kinematic equations and then set to their elevation angle φ_0_. At this point all whiskers now had their correct resting angles 𝜃_0_ and φ_0_ in the coordinate system for the kinematic equations. Finally, all whiskers were rotated by the *negative* of the pitch offset, back into the coordinate system of the morphological model.

Third, we determined the slopes for Δφ/Δ𝜃 and Δζ/Δ𝜃 from Table 1 and Figure 2 in Knutsen et al. (2008). These slopes are in the coordinate system of the kinematic equations. Following a procedure similar to that of step 2, these slopes were converted to the coordinate system of the morphological model.

Fourth, we converted the desired range of protraction angles Δ𝜃 from morphological into kinematic coordinates. We used the slope relationships Δφ/Δ𝜃 and Δζ/Δ𝜃 to obtain the full kinematic equations, and then converted the full kinematic equations back into morphological coordinates.

Notably, **Table 1** shows that within the coordinate system of Knutsen et al. (2008), the resting elevation angle φ_0_ is constant within each row. In contrast, after conversion to the coordinate system of the morphological model, each whisker has a unique resting elevation angle φ_0_. In other words, we must assign a specific value of φ_0_ for each individual whisker. The average and standard deviation of these values are provided for each row of whiskers in the bottom half of **Table 1.**

**Table 1** also shows that after coordinate conversion the equations that define the slope of φ with respect to 𝜃 (Δφ/Δ𝜃) and the slope of ζ with respect to 𝜃 (Δζ/Δ𝜃) varied for each whisker independently. However, the slopes were found to be similar enough across all whiskers within a row that a single average value was used on a per-row basis.

Comparing results between simulations that used the original kinematic equations with those that used the coordinate-transformed kinematic equations resulted in significant shifts at the level of individual whiskers. However, the coordinate transformation did not generally change the trends across the array (e.g., **Figures 4**, **5**, and **7**) or significantly affect uniqueness of the mappings (**Table 2**). Thus the results described in the present study are robust to fairly large changes in the kinematic equations for whisker motion. Similar robustness was observed in a simulation study that included a sensitivity analysis of variations in the geometry of whisker-object contact (Hobbs et al., 2016a).

**Table 2:** Number of pegs reached and the percent of the (M_B_, M_D_) → (*x, y*) mapping that is unique.

Whisker	N pegs	% unique
α	535	100.00%
A1	446	100.00%
A2	305	99.99%
A3	166	100.00%
A4	66	100.00%
β	1390	97.42%
B1	1023	98.88%
B2	668	99.93%
B3	397	100.00%
B4	194	100.00%
B5	55	100.00%
γ	1656	99.53%
C1	1048	98.79%
C2	616	98.71%
C3	344	99.20%
C4	179	97.66%
C5	69	95.94%
C6	6	N/A
δ	1090	98.49%
D1	579	98.53%
D2	307	97.72%
D3	173	98.94%
D4	98	100.00%
D5	55	100.00%
D6	12	100.00%
E1	147	97.44%
E2	46	96.00%
E3	44	93.63%
E4	33	92.94%
E5	28	93.75%
E6	12	100.00%


*N pegs: number of pegs; % unique: percent of the (*M*_*B*_,*M*_*D*_) space that uniquely maps to an (*x, y*) peg location. Whisker C6 was simulated but is labeled N/A because it reached too few pegs to form a surface for this analysis.*

### Choice of Protraction Amplitude

We were careful to ensure that each whisker was simulated to protract through a large range of whisking angles (**Figure 1C**). A simulation began with all whiskers retracted until they were tangent to the surface of the rat’s head (left subplot). Each whisker was then simulated to protract through its resting angle (middle subplot) until one of two terminating criteria was met (right subplot): either it reached total protraction amplitude of 120°, or the *y*-coordinate of the whisker-tip started to decrease. For most whiskers, this range exceeded the protraction amplitudes observed in many experimental studies (Carvell and Simons, 1990; Gao et al., 2001, 2003; Towal and Hartmann, 2006; Hill et al., 2008; Towal and Hartmann, 2008; Grant et al., 2009, 2012; Hobbs et al., 2016b). These extreme choices for protraction amplitude ensured that our uniqueness and resolution measurements (*Results* Sections “Most Whiskers Show Unique Mappings” and “Both Elevation and Roll Contribute to the Uniqueness and the Resolution of the Mappings”) covered all regions of the whisking space.

The simulation was run in two stages, using coarse and fine spatial resolution. In the first stage the approximate location of the peg was determined by simulating the protraction over the full range of whisker protraction in increments of 2°. This first stage narrowed the range of possible peg-whisker contact location to an annular sector with an opening angle of 2° and two radii that differed by 2% of the whisker length. The second stage then started from the angle found in stage one, and protracted the whisker forward with a 0.02° step size for up to 100 steps or until the distance between the peg and the nearest point on the whisker stopped decreasing. The whisker was considered to have contacted the peg at the protraction angle when the peg-to-whisker distance was minimal.

To simulate whisker deflection, each whisker was protracted 5° past the angle at which it would have made contact with the peg, had the peg been present. The undeflected 3D shape of the whisker as well as the 3D position of the peg were the two inputs to the quasi-static numerical model described in Section “Computing Mechanical Signals as the Whisker Protracts against a Peg.” All mechanical signals analyzed in *Results* were determined at the end of the 5° protraction.

A value of 5° was chosen for two reasons. First, when we examined the three studies describing the behavioral experiments (Knutsen et al., 2006; Mehta et al., 2007; O’Connor et al., 2010), our best understanding was that the rodents deflected their whiskers against the peg by only a few degrees. Other studies that have explicitly monitored contact durations or angles during object exploration have found similar evidence for light touch (Deutsch et al., 2012; Hobbs et al., 2015), and light touch is also observed during detection and orienting behaviors (Grant et al., 2009; Mitchinson et al., 2011). Second, the whisker tends to slip off the peg if it protracts too far (Williams and Kramer, 2010; Hires et al., 2013).

### Coordinate Systems: Whisker-Centered vs. Head Centered Coordinates

**FIGURE 2:**
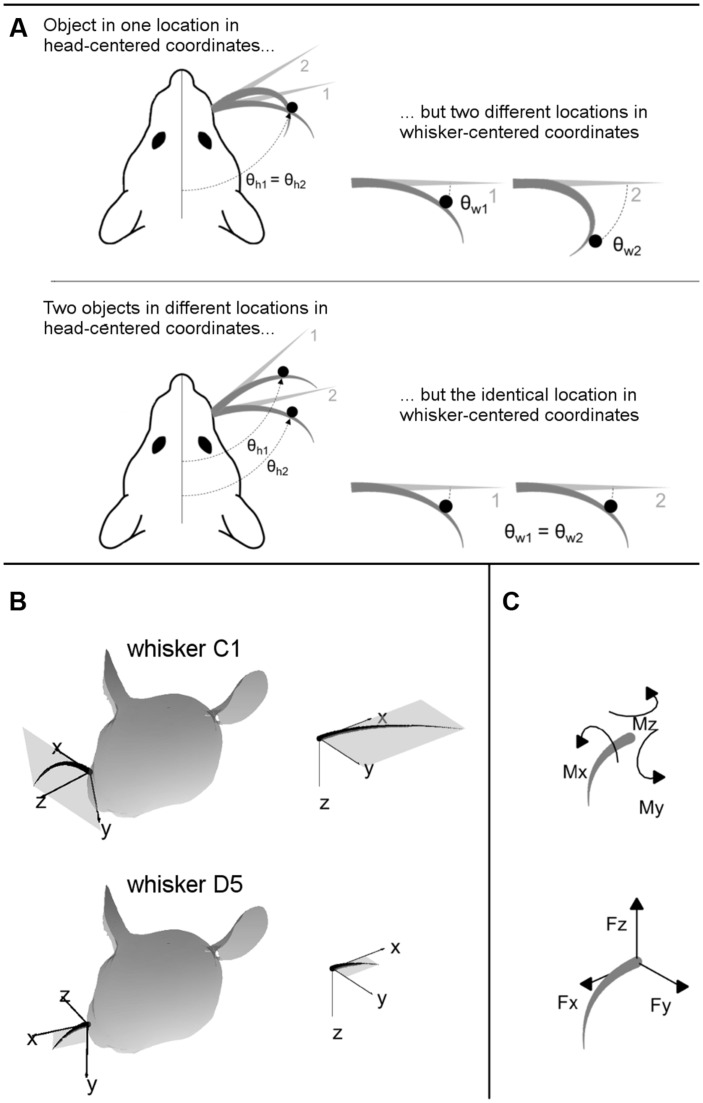
**(A)** 2D intuition for head-centered and whisker-centered coordinate systems. The origin for head centered coordinates is typically taken to be the tip of the snout, with the *y*-axis pointing rostrally along the midline. In contrast, the origin for whisker centered coordinates is at the whisker base, and the *x*- and *y*- axes are determined by the plane in which the whisker lies. The differences between these two coordinate systems are exemplified in two extreme cases, as labeled. Although the rat orients to objects in head-centered coordinates, the only tactile information available is most naturally represented in whisker-centered coordinates. **(B)** 3D intuition for head-centered and whisker-centered coordinate systems. In all panels gray trapezoids indicate the plane of the whisker. (Left panels) Whiskers C1 and D5 are illustrated in their resting positions on the rat’s face. Whisker-centered coordinates are indicated. (Right panels) Whiskers C1 and D5, still in their whisker-centered coordinate systems, are rotated to the same 3D angle to facilitate visual comparison. **(C)** Mechanical signals at the whisker base. When a whisker is deflected by an external point load it generates three forces and three moments at the whisker base.

Understanding the nature of vibrissal-based object localization requires making a clear distinction between the two coordinate systems illustrated in **Figure 2**: head-centered coordinates and whisker-centered coordinates.

Orienting behavior is expressed by motion of the rat’s entire head. In head-centered coordinates the location of an object is most easily expressed using the snout as the origin. The rat must be able to orient its snout toward an object regardless of which whisker(s) made contact with the object.

However, each whisker in the array has a unique arc length and a unique intrinsic curvature. Each whisker has a different basepoint on the rat’s cheek, and each whisker emerges from the cheek with a different set of angles when at biomechanical rest. Each whisker is rotated by a different intrinsic (“sling”) muscle, which wraps around the follicle at its base.

The important consequence of this geometry, as illustrated in two dimensions in **Figure 2A**, is that the tactile (mechanical) signals that enter the follicle due to contact with an object are obtained in whisker-centered coordinates, which rotate with each whisker (Hartmann, 2015; Huet et al., 2015; Huet and Hartmann, 2016). Tactile information depends only on the difference between the shape of the whisker before and after deformation. The position of the whisker before deformation is not directly encoded in the transmitted signals, and there are few if any proprioceptors in the whisker muscles to tell the rat where the whisker is in head centered coordinates (Moore et al., 2015).

**Figure 2B** provides intuition for the 3D transformations between whisker-centered and head-centered reference frames. In this figure, whiskers C1 and D5 are shown in their resting positions on the rat’s face, in the context of their two unique, whisker-centered coordinate systems. The orientation of the C1 whisker is primarily concave down, so the *x–y* axes form nearly a vertical plane while the *z*-axis points mostly forward. The orientation of the D5 whisker is primarily concave forward, so the plane formed by the *x*–*y* axes is more horizontal (tilted out of the page in **Figure 2B**) while the *z*-axis points forward and a bit up. Although the coordinate systems for the C1 and D5 whiskers may initially appear very different in the context of the rat’s head, it is clear from the right panels of **Figure 2B** that they are identical whisker-centered coordinate systems.

The specific question addressed by the present work is: are the tactile signals obtained by any single whisker and transmitted to the follicle – in *whisker*-centered coordinates – sufficient to uniquely determine the 2D location of an object in *head*-centered coordinates?

### Computing Mechanical Signals as the Whisker Protracts against a Peg

The present work uses a quasi-static model for whisker bending that permits us to simulate 3D mechanics and the whisker’s slip along a peg (Huet et al., 2015; Huet and Hartmann, 2016).

Details of the model have been described in these earlier studies, but briefly, each whisker is simulated as a 100-link Euler-Bernoulli beam connected by linear and torsional springs. Whiskers were simulated to have a Young’s modulus of 3 GPa (Quist et al., 2011). Whisker arc lengths (*S*) were determined based on their (row, column) position within the array (Towal et al., 2011). Base diameters (*D_base_*) were calculated based on the linear relationship *D_base_* = 0.07113 + 0.00208*S*, where *S* and *D_base_* are expressed in millimeters (Belli et al., in revision). Whisker tips are often damaged, so tip diameter can vary considerably. Tip diameter was therefore assigned a constant value of 4.1 μm, calculated as the average across 52 whiskers (Belli et al., in revision).

The inputs to the model are the 3D location of the peg, and the 3D intrinsic position and shape of the undeflected whisker, protracted 5° past the angle at which it would have made contact with the peg. The outputs of the model are the deflected whisker shape and the resultant mechanical signals at the whisker base. The mechanical signals can be written in terms of six components illustrated in **Figure 2C**: three components of force (F_x_, F_y_, and F_z_) and three components of moment (M_x_, M_y_, and M_z_).

The physical meaning of each component is as follows: F_x_ is the axial force, which acts along the direction of whisker shaft, pushing or pulling the whisker directly into or out of the follicle. F_y_ and F_z_ are the transverse forces, which act along the *y*- and *z*- axes in whisker-centered coordinates, respectively. F_y_ increases or decreases the curvature of whisker depending on its sign, while F_z_ bends the whisker in and out of the plane of its intrinsic curvature. The torsional moment, M_x_, twists the whisker about its own shaft. The bending moments M_y_ and M_z_ are correlated, respectively, with F_z_ and F_y_ by the radial distance of contact point from the base of whisker.

We reiterate that the present work assumes that rodents receive mechanical signals from individual whiskers without knowledge of the angles of the whisker in head-centered coordinates.

### The “Average Minimum Distance” as Metric to Quantify the Resolution of the Mappings

Some analyses (**Figures 5–7**) required a metric to quantify the resolution of the mapping between mechanical signals at the whisker base and the (*x, y*) location of the peg. The idea here is that a given combination of mechanical signals should map to a single (*x, y*) location of a peg in head-centered coordinates. Furthermore, the (*x, y*) location of the peg predicted by those mechanical signals should be far away from the (*x, y*) locations of any other peg.

We therefore defined the “average minimum distance,” as follows: first, because the bending magnitude and bending direction (*M*_B_ and *M*_D_, respectively; see *Results* for calculation) have different dimensions and different orders of magnitudes (10^-6^ and 10^0^), they were normalized between 0 and 1. Then, for each data point (*M*_B,n_, *M*_D,n_) we calculated the distance D_nm_ from every other (*M*_B,m_, *M*_D,m_) point in the dataset: $D_{nm}=\sqrt{{\left(M_{B,m}-M_{B,n}\right)}^{2}-{\left(M_{D,m}-M_{D,n}\right)}^{2}}$. The point (*M*_B,m_, *M*_D,m_) with the minimum value of D_nm_ was denoted as D_n,min_ and was selected as the nearest neighbor to (*M*_B,n_, *M*_D,n_). The same procedure was performed for all *N* data points in the entire mapping. The sum of all minimum distances $\Sigma_{n=1}^{N}D_{n,\min}$ divided by the total number of data points *N* defined the average minimum distance. Intuitively, this metric provides a measure that answers the question “on average, how close is the nearest (*M*_B_, M_D_) neighbor?”

## Results

### Mechanical Signals at the Whisker Base in Whisker-Centered Coordinates Vary with the Peg’s Location in Head-Centered Coordinates

**FIGURE 3:**
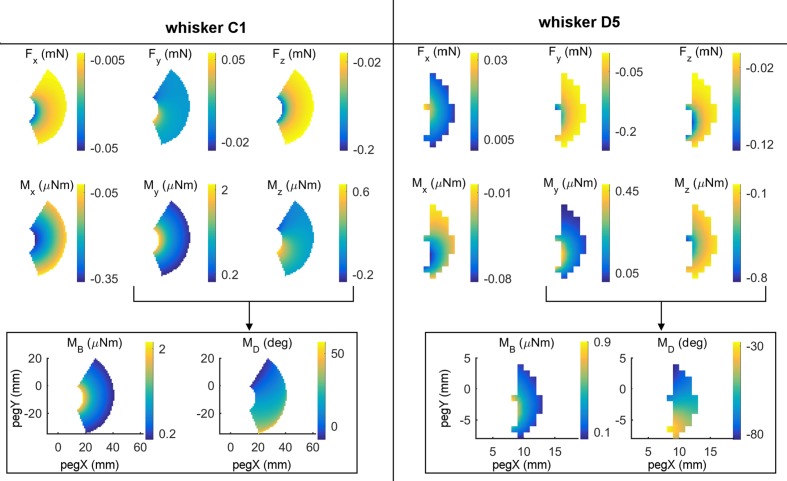
**Mechanical signals vary with the (*x, y*) location of contact.** The left and right halves of this figure display results for two different whiskers: C1 and D5. **(Top two rows)** All six components (F_x_, F_y_, F_z_, M_x_, M_y_, and M_z_) of mechanical signals after the whisker was simulated to deflect 5° against pegs at different *x, y* locations in head-centered coordinates. The location (0,0) represents the rat’s snout. At any *x, y* location in each subplot, color represents the magnitude of the signal at the whisker base after the 5° deflection against the peg at that *x, y* location. Colors were interpolated between peg locations and the relevant scale for each signal is shown in the colorbars. **(Bottom row)** The signals M_y_ and M_z_ can be combined into the magnitude and direction of the bending moment. Notice that for whisker C1, M_B_ almost entirely follows M_y_ because M_z_ is so much smaller than M_y_. The same is not true for D5 because it pushes differently against the peg.

All six components of force and moment at the whisker base were found to exhibit systematic variations with the (x,y) location of the peg in head centered coordinates. Examples of these variations are shown in the first two rows of subplots in **Figure 3** for whiskers C1 and D5. For each whisker, four of the six mechanical signals vary primarily as a function of the radial distance to the peg. For the C1 whisker these four signals are F_x_, M_x_, F_z_, and M_y_, while for the D5 whisker the four signals are F_x_, M_x_, F_y_, and M_z_. However, one of the forces and its corresponding moment have different signs and magnitudes when the whisker presses against posterior compared to anterior pegs. These signals are F_y_ and M_z_ for C1 and F_z_ and M_y_ for D5. These signals therefore exhibit significant variation in a posterior/anterior direction.

The near orthogonality of the two types of variations suggests that there may be multiple combinations of mechanical variables that could uniquely represent the two parameters (*x, y*) that define the location of the peg. Rather than try every possible combination of the raw signals one by one, we turned to neurophysiological results for guidance.

Recordings from primary sensory neurons in the trigeminal ganglion show that responses correlate strongly with both the magnitude (Szwed et al., 2003, 2006) and direction (Simons, 1978, 1985; Gibson and Welker, 1983; Lichtenstein et al., 1990; Jones et al., 2004; Lottem and Azouz, 2011; Lottem et al., 2015) of whisker bending. We therefore rewrote the *y*- and *z*- components of the bending moments in terms of the magnitude and direction of their vector sum: $M_{B}=\sqrt{M_{y}^{2}+M_{z}^{2}}$ and $M_{D}=a\tan\;\left(\frac{M_{z}}{M_{y}}\right)$.

As shown in the bottom row of **Figure 3**, the mappings for M_B_ and M_D_ are excellent complements in that M_B_ is mostly correlated with radial distance, while M_D_ is more correlated with the angular location of contact. In the next two sections we therefore ask first, whether these two signals vary systematically with peg location, and second, to what extent these two signals alone can uniquely represent the (*x, y*) location of the peg.

### Bending Magnitude and Direction Vary with Peg Location for All Whiskers

**FIGURE 4:**
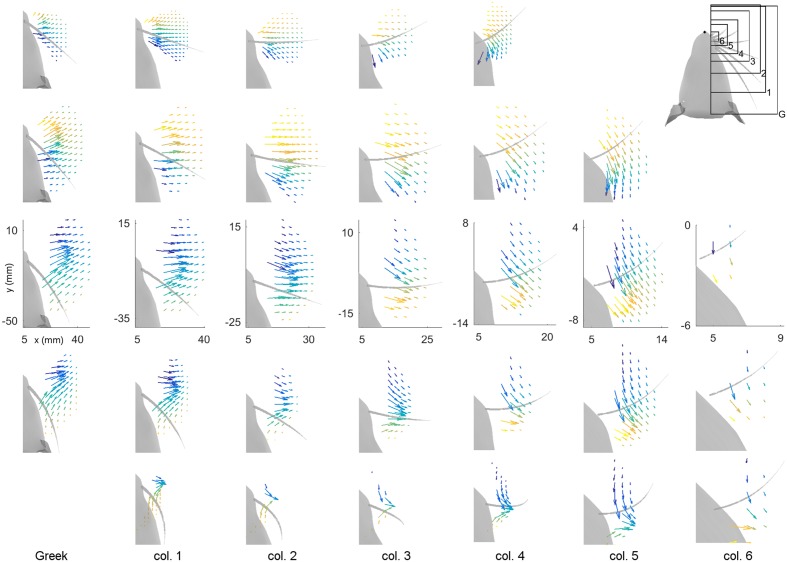
**Magnitude and direction of bending moment at the whisker base after a 5° rotation against pegs placed at different (*x, y*) locations relative to the rat’s head.** The subplots in this figure are positioned in the same pattern as the whiskers on the mystacial pad of the rat, from rows A through E (top–down) and columns Greek through 6 (left-right). Each subplot shows the resting position and orientation of that whisker as a gray, tapered curve. The axes represent the (*x, y*) locations of the pegs, with the origin at the rat’s snout. Colored vectors represent the magnitude and direction of the bending moment for that whisker. The color of the vector also represents bending direction because it is difficult to see the direction of some of the very short vectors. Each vector has its origin at the peg location. All subplots within a single column are on the same scale. The subplots in different columns are on different scales because the whiskers differ greatly in length between columns. Axes are shown only within the C-row to indicate that the identical scales apply within each column. The upper-right subplot is a reference for the scales of the plots in each column. Both M_B_ and M_D_ are scaled identically within each column, but they are scaled differently between columns. All simulations were run with 1 mm resolution in both *x*- and *y*-, but the number of pegs in each subplot has been reduced for visual clarity.

To represent M_B_ and M_D_ intuitively within a single plot, vector fields are used to represent these two variables at each peg location for all 31 whiskers of the array. Results are shown in **Figure 4.** In this figure the subplots are spatially arranged to match the arrangement of the vibrissae on the rat’s face. Each subplot shows the vector field of bending magnitude and direction for one whisker. The origin of each vector is placed at the (*x, y*) location of the peg of contact. The magnitude of the vector represents the magnitude of the bending moment, and the direction of the vector indicates the direction of the bending moment. Because it is difficult to see the direction of some of the shorter vectors, the bending direction is also represented by the color of each vector. We emphasize that although the figure illustrates the direction of bending moment in the (*x, y*) plane, the direction of bending moment is actually a combination of the two bending moments in the *y*- and *z*- directions in whisker-centered coordinates. Both *y*- and *z*- axes are perpendicular (transverse) to the whisker shaft.

The first feature evident in **Figure 4** is that each whisker reaches very different numbers and very different subsets of pegs. The subplots are on the same scale within each column so the size of the regions reached can be directly compared (from A row to B row, etc.…). The scale of the subplots changes between columns, but the shapes of the regions that can be reached can be compared from column to column (e.g., from Column 1, to 2, to 3, etc.…). For example, rostral whiskers were able to reach more anterior pegs; there is more variability between rows than between columns; and in general, whiskers in rows B and C reach the most pegs.

The second result revealed by **Figure 4** is that both the magnitude and the direction of the bending moment vary systematically for each of the 31 whiskers. As suggested by the plots of **Figure 3**, the direction of bending varies primarily with the peg’s anterior/posterior location, while the magnitude of bending varies more with radial distance. Moreover, there is a large degree of variability from whisker to whisker, even for two whiskers close together, for example β and γ. The question remains, however, to what extent the vector plots shown in **Figure 4** represent unique mappings to the (*x, y*) locations of the pegs.

### Most Whiskers Show Unique Mappings

**FIGURE 5:**
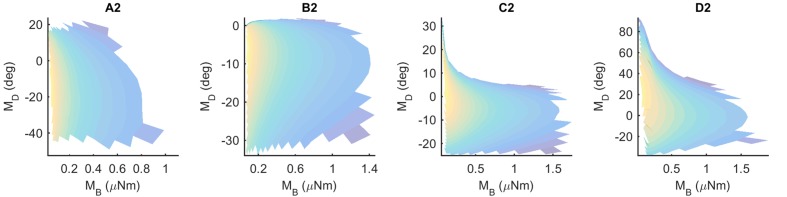
**Mappings for the Column-2 whiskers are generally unique, typical of all whiskers of the array.** The *x*- and *y*- axes represent the magnitude and direction of bending moment, respectively. All data points are interpolated to form a surface colored by the x-locations of the pegs. The surface has been rendered to be partially transparent, so that non-unique (overlapping) regions of the mappings are revealed. Overlaps, if present, are visible as regions of high color intensity. In this example of the column 2 whiskers, whisker B2 is non-unique in the extremely narrow dark blue strip near *M*_D_ = 0. Whisker D2 has tiny non-unique patches with steep negative slopes near *M*_B_ = 0.30 and *M*_D_ = 60. Overlaps would remain the same for all whiskers if they were colored by the *y*-location of the pegs instead of the *x*-location.

If a mapping between (M_B_,M_D_) and (x,y) is unique, then each pair (M_B_,M_D_) should point to only one x value and only one *y* value. These mappings can be visualized using the conventions shown in **Figure 5**, which shows results for the Column 2 whiskers. In each of the subplots, M_B_ is represented on the *x*-axis and M_D_ is represented on the *y*-axis. Each point on the plot is assigned a color corresponding to the associated *x*- or *y*-location of the peg. Points are interpolated between locations, and the plot is made transparent so that uniqueness can be directly visualized. Non-unique locations in the map show sharp edges of brightened color where the surface “folds over” on itself. The “percent uniqueness” (**Table 2**; **Figure 5**) was quantified as the percent of surface plot area that did not contain overlap.

The results in **Figure 5** indicate that the mappings are nearly completely analytically unique for all whiskers of column 2. There is an extremely narrow band of overlap for the B2 whisker where M_D_ is close to zero, and for patches of overlap of the D2 whisker in the top left corner, where *M*_D_ is large and *M*_B_ is small.

These results were typical for all columns of whiskers, as summarized in **Table 2.** Of the 31 whiskers tested in the array, 30 reached sufficient number of pegs to generate surface plots that could be analyzed for uniqueness (the exception was whisker C6, which reached very few pegs). Of the 30 surface plots, 11 gave completely (100%) unique results, ten were more than 98% unique, six were more than 95% unique, and the remaining three were more than 92% unique. It is worth noting that all three whiskers with less than 95% uniqueness belonged to row E: whiskers in this row reach far fewer pegs than the other rows (c.f., **Figure 4**), and the orientation of whiskers is more concave-downward.

### Both Elevation and Roll Contribute to the Uniqueness and the Resolution of the Mappings

**FIGURE 6:**
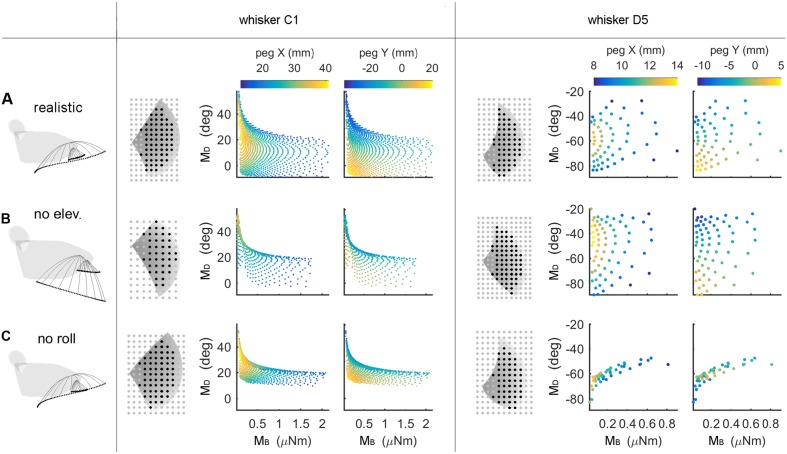
**Mappings between bending magnitude and direction (M_B_,M_D_) and peg location (*x, y*) as whisking kinematics are altered.** Each row of the figure shows data from the corresponding row of **Table 3.** The first figurine in each row shows a side view of the kinematic profile of the C1 and D5 whiskers. Solid gray lines are representative whisker shapes in 10° increments, and black dots are the whisker tip positions. The grayscale plots in each row show the pegs that can be reached by the whisker (black dots) or not (gray dots). The grayscale shading indicates the *z*-coordinate (height) of the whisker’s trajectory, and the pegs for the C1 whisker are four times denser than shown in the plot. The colored subplots in each row show the mappings between bending magnitude and direction (M_B_,M_D_) and the (*x, y*) location of the peg. Colormaps are the same for all subplots within each column, as indicated in the colorbars at the top of each column. All colored plots for each whisker have the same axis limits **(A)** Normal whisking kinematics. **(B)** No elevation. **(C)** No roll.

An intrinsic limitation of the type of simulation results shown in **Figure 5** is that a solution could be declared “unique” even if two mappings differed by only floating point error. Uniqueness does not have full practical utility unless a measure of resolution is added. Therefore, the “average minimum distance” (AMD, see Materials and Methods) was used to evaluate the resolution of each of the mappings. The C1 and D5 whiskers are used as typical, illustrative examples in **Figure 6**, but analysis was run on all whiskers in the array.

The colored subplots in **Figure 6** are similar to those in **Figure 5**, except that the data points have not been interpolated to form a surface. The color of each data point corresponds to the peg’s *x*- or *y*-location, and differentiation between peg locations is associated with larger distances between data points. It is clear that for whisker C1 in **Figure 6A**, the data points are spread out and that peg locations vary continuously. Whisker D5 shows a similar trend but reaches fewer pegs (it is a shorter whisker), so the data points are further apart from each other.

We next examined the effect of features of whisking kinematics on the mappings. As described in Section “Materials and Methods,” when the vibrissal array protracts, each whisker exhibits a small amount of dorsoventral elevation (Bermejo et al., 2002; Knutsen et al., 2008; Knutsen, 2015) as well as roll about its own axis (Knutsen et al., 2008; Knutsen, 2015). We performed simulations with elevation and roll removed, as illustrated in **Figures 6B,C.** Qualitatively, these figures demonstrate that when either roll or elevation is removed, the data points distribute across a smaller region of the (M_B_,M_D_) space, meaning that the mapping resolution deteriorates. In addition, for both whiskers C1 and D5, removal of either roll or elevation altered the total number of pegs reached.

The effects of removing elevation are shown in **Figure 6B** and in **Table 3.** For long, caudal whiskers such as C1, removing elevation decreases the range of bending magnitude (*M*_B_) by approximately 20%, and the range of bending direction (*M*_D_) decreases even more. These effects are quantified in the middle row of **Table 3**, which indicates that resolution (as measured by AMD) decreases to 69% of its value measured during normal kinematics. For short, rostral whiskers such as D5, the effect of removing elevation is more difficult to observe. Only small differences are visible in **Figure 6B.** **Table 3** shows that quantitatively, the AMD decreased by ∼6%, but uniqueness was unaffected and the number of pegs reached actually increased. Thus, by comparing effects between whiskers C1 and D5, we see that the effect of elevation is strongly influenced by the particular whisker’s angle of emergence and the orientation of the whisker’s trajectory with respect to the peg.

**Table 3:** The effect of elevation and roll on the number of pegs reached and the (M_B_, M_D_) → (*x, y*) mapping uniqueness and resolution.

	Whisker C1			Whisker D5		
		
	N pegs	% unique	AMD	N pegs	% unique	AMD
Realistic kinematics, including roll and elevation	1048	98.79%	1	55	100.00%	1
No elevation	633	96.28%	0.691	67	100.00%	0.940
No roll	976	82.21%	0.522	50	51.75%	0.339


**N pegs*: number of pegs; *% unique*: percent of the (*M*_*B*_,*M*_*D*_) space that uniquely maps to an (*x, y*) peg location; *AMD*: average minimum distance computed over the entire mapping. The AMD has been normalized to equal exactly 1 for normal whisking kinematics so that the effects of altered kinematics can be quantified.*

Removing roll has a more consistent effect across whiskers, as shown in **Figure 6C** and the bottom row of **Table 3.** The range of bending direction (*M*_D_) is dramatically reduced for both whiskers C1 and D5. Uniqueness also drops significantly for both whiskers (to ∼82% for C1, and to ∼52% for D5), and mapping resolution is reduced to AMD = 0.52 and AMD = 0.34 for the two whiskers.

It is interesting to note that the removal of either elevation or roll has a much larger effect on bending direction than on bending magnitude. The change in bending magnitude is dominated by the radial distance from the contact point to the whisker base for a fixed pushing angle (in this case, 5°).

Summarizing, both roll and elevation contribute in important ways to the quality of mappings, but in different respects. Roll consistently increases both mapping resolution and uniqueness by a significant amount. Elevation contributes to resolution and may or may not to uniqueness, depending on whisker identity.

### Localization of a Peg Using a Row vs. a Column of Whiskers

We can now use the mappings found in the preceding sections to suggest explanations for some results of the behavioral studies (Knutsen et al., 2006; Mehta et al., 2007; O’Connor et al., 2010). In all three experiments rodents were required to indicate the horizontal (anterior/posterior) location of a vertical peg. All three studies found that animals could perform the task if whiskers were trimmed such that only one row of whiskers remained (the C row), or even if only one whisker remained (typically C1 or C2), albeit at a reduced performance level.

A particularly intriguing result of one of the studies (Knutsen et al., 2006) was that rats obtained the highest localization acuity (<1.5 mm) if they were initially trained on the task with all whiskers intact, and then trimmed to leave only one column (instead of a row) of whiskers.

To explain this result, we hypothesized that, for pegs placed in different anterior/posterior locations, the mappings between (M_B_,M_D_) and the (r, 𝜃) positions of the pegs in head-centered coordinates should be more different for a column of whiskers than for a row of whiskers.

**FIGURE 7:**
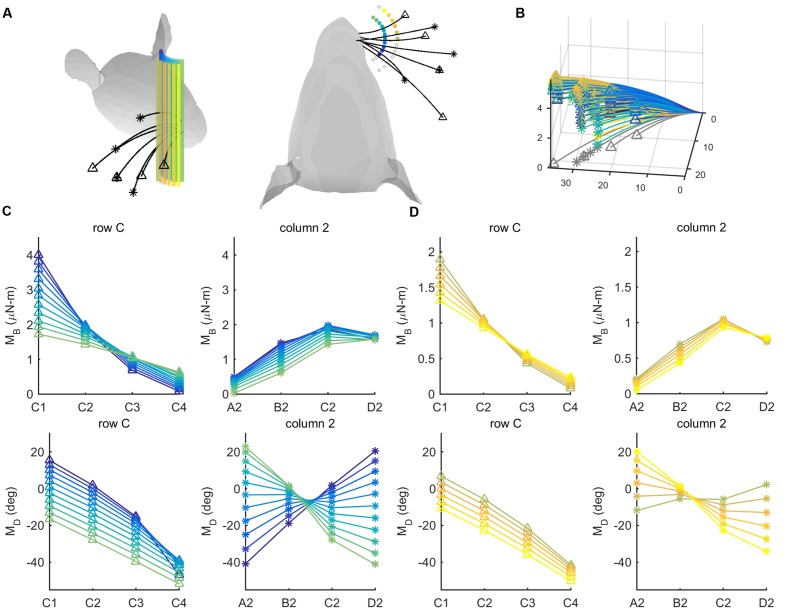
**Mechanical signals within rows and columns of whiskers.** **(A)** 26 pegs (colored cylinders) are placed at radial distances *r* = 8 and *r* = 12 mm from the average location of the whisker base points. The pegs are spread from -60° to 60° in 10° increments in head-centered coordinates. Four whiskers of the C row (C1–C4, tips marked with triangles) and four whiskers of column 2 (A2-D2, tips marked with asterisks) are simulated to protract 5° against the peg. **(B)** The same whiskers as in **(A)** are now shown in whisker-centered coordinates. All undeflected whiskers lie in the *x–y* plane, and all proximal segments align with the *x*-axis. Whiskers are shown in gray before deflection and in color after deflection. The colors of the deflected whiskers correspond to the pegs in panel **(A)**. **(C)** The four subplots show bending magnitude and bending direction for pegs placed at a radial distance *r* = 8 mm from the average whisker base. Bending magnitudes and bending directions are plotted for the C-row (triangles) and Column-2 (asterisks) whiskers. The color code corresponds to the peg locations shown in panel **(A)**. **(D)** The same subplots as in **(C)**, but for pegs placed at a radial distance *r* = 12 mm from the average whisker base. The color code again corresponds to the peg locations shown in panel **(A)**.

We tested this hypothesis by simulating deflections of the C-row and column-2 whiskers against a set of 26 pegs, as shown in **Figures 7A,B.** These are the same whiskers used in the behavioral experiment of Knutsen et al. (2006). The pegs were placed at radial distances either 8 or 12 mm from the average location of the whisker basepoints. The pegs were distributed from -60° to 60° in 10° increments, yielding 13 pegs at each of the two radial distances. Results, shown in **Figures 7C,D**, lend support to our hypothesis.

For both proximal and distal radial distances (**Figures 7C,D**), the curves for bending magnitude (*M*_B_) are similar across all whiskers in the C-row: *M*_B_ monotonically decreases from C1 to C4. The *M*_B_ curves are also quite similar across the column-2 whiskers: *M*_B_ increases from A2 to C2, and then tends to decrease for whisker D2. The curves for bending direction (*M*_D_) are also similar across all whiskers in the C-row: *M*_D_ decreases from C1 to C4.

The curves for *M*_D_ across the column-2 whiskers, however, exhibit very different slopes depending on the posterior/anterior location of the peg. The A2 whisker will be oriented slightly concave-forward when it makes contact with a peg at a posterior location. Contact with the peg will therefore tend to push the A2 whisker to become straight relative to its intrinsic curvature, resulting in a negative *M*_D_ (dark purple curve in the bottom right subplot in **Figure 7C**). In contrast, the D2 whisker will be oriented slightly concave-backward when it makes contact with a peg at a posterior location. Contact with the peg will tend to push the D2 whisker to curve increasingly in the direction of its intrinsic curvature, resulting in a positive *M*_D_ (light green curve in the bottom right subplot in **Figure 7C**).

Conversely, when the whiskers hit a peg at a more anterior location, the A2 whisker has rolled to become oriented slightly concave-backward, while the D2 whisker has rolled to become oriented more concave-forward, thus the trends are opposite.

Summarizing, pegs at different angular locations can be more easily distinguished from each other on the basis of differences in bending direction within a column, rather than within a row.

## Discussion

How is it possible for a rodent to orient to an object based on tactile information from a single whisker? To date, this has been a challenging question to answer because the 3D tactile signals available to the rat could not be quantified. The present study exploited a 3D model recently developed by our laboratory (Huet et al., 2015; Huet and Hartmann, 2016) to simulate the mechanical signals the rat will obtain as it whisks against a vertical peg. We find that for nearly all whiskers, sufficient information is available in the bending moment signal to localize the peg, provided that the whisker’s kinematic trajectory includes both roll and elevation and the rat’s head exhibits minimal pitch. These constraints are compatible with the conditions observed in three behavioral studies (Knutsen et al., 2006; Mehta et al., 2007; O’Connor et al., 2010).

### Cues for Orienting to an Object

The present work has demonstrated one possible mechanism by which the rat could localize a vertical peg in head-centered coordinates based entirely on tactile information from a single vibrissa. We do not suggest that the mechanism demonstrated here is the only method by which the rat might determine the peg’s location. The nervous system generally exploits all available information to solve perceptual tasks, and multiple redundant cues are likely used.

A complementary possibility is that the rodent could estimate the location of the peg by combining a contact signal with an estimate of the position of the whisker at time of contact. The position signal could be obtained by combining reafferent signals representing the phase of the whisker (Fee et al., 1997; Mehta et al., 2007; Moore et al., 2015; Wallach et al., 2016) with an efference copy of whisking midpoint and amplitude (Curtis and Kleinfeld, 2009; Kleinfeld and Deschênes, 2011). Kinematic (position) control is a viable motor control strategy during non-contact whisking because the whiskers have such low mass; upon contact it seems likely that the rat will shift to a force control strategy (Quist et al., 2014; Hobbs et al., 2016b).

Alternatively, the animal could determine the horizontal angle of a peg by keeping track of the time at which each whisker starts protraction and then integrating whisker velocity with respect to time to obtain an estimate of the whisker’s position at the time of contact (Szwed et al., 2003, 2006; Knutsen et al., 2006; Knutsen and Ahissar, 2009; Huet et al., 2015). This mechanism would require the rat to keep track of the velocity of each whisker with high precision (Towal and Hartmann, 2006, 2008; Grant et al., 2009; Hartmann, 2009; Hobbs et al., 2016b).

Peg localization could also be aided by skin stretch, which could serve in a proprioceptive capacity to provide cues about protraction angle. Weak proprioceptive signals from the muscles could also be carried through the mesencephalic nucleus (Mameli et al., 2010, 2016).

Finally, we note that in order to replicate as faithfully as possible the three previous behavioral experiments, the simulations here used “infinitely tall” vertical pegs. Therefore the present study does not address the question of how the rat might determine the height of an object. As indicated by several previous articles, the height could be at least partially determined by a labeled line system that is determined by the whisker’s identity (Knutsen et al., 2008). Alternatively, use of one or more additional mechanical signals (e.g., the axial force) seems likely to resolve this third coordinate (Solomon and Hartmann, 2011; Pammer et al., 2013).

In our view, it would make excellent sense for timing information coupled with velocity integration to provide a coarse estimate of the angular position of the whisker. At each point in time, the position estimate could be updated with information from skin stretch, weak proprioceptive signals, and efference copy. The final cue about the horizontal angle of contact would emerge at the instant of contact, as the whisker deflects against the object in a direction related to the object’s angular position. The overall location and shape of an object spanning many whiskers could then be coded by tactile feedback from many whiskers integrated simultaneously.

### Potential Mechanisms for Neural Coding

How would mechanical signals transmitted by the whisker be transduced into neural signals while maintaining information about object location? Multiple studies have demonstrated that primary sensory neurons of the trigeminal ganglion are strongly directionally tuned (Simons, 1978, 1985; Gibson and Welker, 1983; Lichtenstein et al., 1990; Jones et al., 2004; Lottem and Azouz, 2011; Lottem et al., 2015). This tuning is related, at least in part, to the angular location of mechanoreceptors within the follicle (Ebara et al., 2002; Knutsen et al., 2008; Rutlin et al., 2014; Whiteley et al., 2015). The bending direction (*M*_D_) could therefore be represented by the identity of mechanoreceptors at particular angular locations within the follicle, resulting in a labeled line code (Knutsen et al., 2008) that represents the azimuthal coordinate of object contact.

Trigeminal ganglion neurons are also well known to respond strongly to the magnitude of whisker deflection (Zucker and Welker, 1969; Gibson and Welker, 1983; Szwed et al., 2003, 2006; Jones et al., 2004). The bending magnitude signal (*M*_B_) is therefore likely to be represented as the number of spikes per unit time as the whisker increasingly deflects against an object, consistent with a role in determining radial distance (Szwed et al., 2003, 2006).

Importantly, the mechanism for object localization proposed in the current work does not depend on whisking velocity, nor on the velocity of the whisker at time of impact, nor on how rapidly or slowly the whisker deflects against the peg. The peg location can be computed for every combination of *M*_B_ and *M*_D_ at each point in time. Thus, with the assumption that friction is negligible (see next section), the animal can determine the contact point location regardless of the time-history of the whisker.

In addition, although the present work simulated only protraction against the peg, symmetry arguments suggest that similar results would hold during retraction. The direction of whisker bending (*M*_D_) is determined by its orientation relative to the peg. This orientation depends on the angles through which the whisker rotates during protraction. Although retraction is unlikely to follow the exact “inverse trajectory” of protraction, it is clear that retraction must ultimately invert all the protraction angles as the whisker returns to its original starting position. Thus the inverted retraction angles will likely exhibit similar uniqueness characteristics as the forward protraction angles.

### Effects of Variable Whisking Kinematics and Friction

In the awake, freely moving animal, whisking kinematics will be more complicated than the 3D trajectories simulated here. In particular, a well-known feature of natural whisking is that the basepoints of the whiskers translate significantly (Knutsen et al., 2008; O’Connor et al., 2010; Knutsen, 2015). Because equations for translational motions have not yet been established, the present work simulated pure rotation and neglected translation.

This simplification is appropriate for two reasons. First, in our own work on body restrained rats, we found that the purely rotational equations provided by (Knutsen et al., 2008) were a remarkably good fit to behavioral data (Huet and Hartmann, 2016). Second, a sensitivity analysis that examined the effect of variations in whisking kinematics on the angle of contact against a surface showed that the effects are systematic across the array (Hobbs et al., 2015, 2016a). Thus, if whisking kinematics are altered slightly, the mappings shown in **Figures 4–6** will exhibit systematic shifts, but will not become degenerate.

Friction will also play a significant role in determining the direction in which the whiskers are deflected after contact (Solomon and Hartmann, 2008, 2010; Boubenec et al., 2012; Pammer et al., 2013; Huet and Hartmann, 2016). The present simulations assumed frictionless conditions, which in general will ensure the maximum vertical deflection along the peg over the course of the 5° protraction (Solomon and Hartmann, 2008, 2010; Huet et al., 2015; Huet and Hartmann, 2016). Friction will influence all mechanical parameters at the whisker base and is likely to introduce non-linear effects into the mappings; this is an important area for future work.

### The Effects of Head Pitch and 3D Mappings in Whisker-Centered Coordinates

As stated above, the present work does not address the question of what happens when the rat pitches its head. Preliminary simulations (not shown) suggest that the mappings will shift but generally retain their uniqueness. For example, in the present work, with bregma aligned with lambda, several whiskers of the C-row showed slightly non-unique mappings. When the head is pitched upward, whiskers of the C-row take on an orientation similar to that which the B-row whiskers had previously. This suggests that the C-row whiskers would increase their uniqueness to match the B-row whiskers before them. The B-row whiskers, in turn, would be oriented more like the A-row whiskers.

In addition, the uniqueness of the present mappings is robust to sizable variations in whisking kinematics. As shown in **Table 1**, all simulations were first run with the kinematic equations of Knutsen et al. (2008), and then re-run after shifting these kinematic equations to the coordinate system of the morphological model (Towal et al., 2011). Although the mappings for individual whiskers varied to some degree, overall uniqueness was not strongly affected.

We also note that the mappings of the present work exploit only two of six possible mechanical variables (My and Mz). It seems likely that a third variable such as the axial force (Fx) or the twisting moment (Mx) could enable unique mappings across head pitch.

Finally, the present work has addressed only the mappings between single whisker contact and the location of a peg in head centered coordinates. A key unresolved issue is the extent to which mechanical signals at the whisker base can represent the 3D location of an object in whisker-centered-coordinates, including the height. If the 3D object-contact location can be determined based purely on tactile signals, then integration of this information across whiskers would permit the animal to obtain an impression of the object’s contour or shape.

## Author Contributions

AY and MH developed the simulations and AY performed the mechanical modeling and simulations. AY and MH analyzed simulation results. AY and MH wrote the manuscript.

## Conflict of Interest Statement

The authors declare that the research was conducted in the absence of any commercial or financial relationships that could be construed as a potential conflict of interest.

## References

[B1] Bermejo R. Vyas A. Zeigler H. P. (2002). Topography of rodent whisking–I. Two-dimensional monitoring of whisker movements. *Somatosens. Mot. Res.* 19 341–346. 10.1080/0899022021000037809 12590835

[B2] Boubenec Y. Shulz D. E. Debregeas G. (2012). Whisker encoding of mechanical events during active tactile exploration. *Front. Behav. Neurosci.* 6:74 10.3389/fnbeh.2012.00074 PMC3490139 23133410

[B3] Carvell G. E. Simons D. J. (1990). Biometric analyses of vibrissal tactile discrimination in the rat. *J. Neurosci.* 10 2638–2648. 2388081 10.1523/JNEUROSCI.10-08-02638.1990 PMC6570272

[B4] Curtis J. C. Kleinfeld D. (2009). Phase-to-rate transformations encode touch in cortical neurons of a scanning sensorimotor system. *Nat. Neurosci.* 12 492–501. 10.1038/nn.2283 19270688 PMC2863011

[B5] Deutsch D. Pietr M. Knutsen P. M. Ahissar E. Schneidman E. (2012). Fast feedback in active sensing: touch-induced changes to whisker-object interaction. *PLoS ONE* 7:e44272 10.1371/journal.pone.0044272 PMC3445569 23028512

[B6] Ebara S. Kumamoto K. Matsuura T. Mazurkiewicz J. E. Rice F. L. (2002). Similarities and differences in the innervation of mystacial vibrissal follicle-sinus complexes in the rat and cat: a confocal microscopic study. *J. Comp. Neurol.* 449 103–119. 10.1002/cne.10277 12115682

[B7] Fee M. S. Mitra P. P. Kleinfeld D. (1997). Central versus peripheral determinants of patterned spike activity in rat vibrissa cortex during whisking. *J. Neurophysiol.* 78 1144–1149. 9307141 10.1152/jn.1997.78.2.1144

[B8] Gao P. Bermejo R. Zeigler H. P. (2001). Whisker deafferentation and rodent whisking patterns: behavioral evidence for a central pattern generator. *J. Neurosci.* 21 5374–5380. 11438614 10.1523/JNEUROSCI.21-14-05374.2001 PMC6762837

[B9] Gao P. Ploog B. Zeigler H. (2003). Whisking as a “voluntary” response: operant control of whisking parameters and effects of whisker denervation. *Somatosens. Mot. Res.* 20 179–189. 10.1080/08990220310001623031-411 14675957

[B10] Gibson J. M. Welker W. I. (1983). Quantitative studies of stimulus coding in 1st order vibrissa afferents of rats. 2. Adaptation and coding of stimulus parameters. *Somatosens. Res.* 1 95–117. 10.3109/07367228309144543 6679920

[B11] Grant R. A. Mitchinson B. Fox C. W. Prescott T. J. (2009). Active touch sensing in the rat: anticipatory and regulatory control of whisker movements during surface exploration. *J. Neurophysiol.* 101 862–874. 10.1152/jn.90783.2008 19036871 PMC2657060

[B12] Grant R. A. Sperber A. L. Prescott T. J. (2012). The role of orienting in vibrissal touch sensing. *Front. Behav. Neurosci.* 6:39 10.3389/fnbeh.2012.00039 PMC3391677 22787445

[B13] Hartmann M. J. Z. (2009). Active touch, exploratory movements, and sensory prediction. *Integr. Comp. Biol.* 49 681–690. 10.1093/icb/icp107 21665850

[B14] Hartmann M. J. Z. (2015). Vibrissa mechanical properties. *Scholarpedia* 10:6636 10.4249/scholarpedia.6636

[B15] Hill D. N. Bermejo R. Zeigler H. P. Kleinfeld D. (2008). Biomechanics of the vibrissa motor plant in rat: rhythmic whisking consists of triphasic neuromuscular activity. *J. Neurosci.* 28 3438–3455. 10.1523/JNEUROSCI.5008-07.2008 18367610 PMC6670594

[B16] Hill D. N. Curtis J. C. Moore J. D. Kleinfeld D. (2011). Primary motor cortex reports efferent control of vibrissa motion on multiple timescales. *Neuron* 72 344–356. 10.1016/j.neuron.2011.09.020 22017992 PMC3717360

[B17] Hires S. A. Pammer L. Svoboda K. Golomb D. (2013). Tapered whiskers are required for active tactile sensation. *Elife* 2:e01350 10.7554/eLife.01350 PMC3828597 24252879

[B18] Hobbs J. A. Towal R. B. Hartmann M. J. Z. (2015). Probability distributions of whisker-surface contact: quantifying elements of the rat vibrissotactile natural scene. *J. Exp. Biol.* 218 2551–2562. 10.1242/jeb.116186 26290591 PMC6514453

[B19] Hobbs J. A. Towal R. B. Hartmann M. J. Z. (2016a). Evidence for functional groupings of vibrissae across the rodent mystacial pad. *PLoS Comput. Biol.* 12:e1004109 10.1371/journal.pcbi.1004109 PMC4706419 26745501

[B20] Hobbs J. A. Towal R. B. Hartmann M. J. Z. (2016b). Spatiotemporal patterns of contact across the rat vibrissal array during exploratory behavior. *Front. Behav. Neurosci.* 9:356 10.3389/fnbeh.2015.00356 PMC4700281 26778990

[B21] Huet L. A. Hartmann M. J. Z. (2014). The search space of the rat during whisking behavior. *J. Exp. Biol.* 217 3365–3376. 10.1242/jeb.105338 25232200

[B22] Huet L. A. Hartmann M. J. Z. (2016). Simulations of a vibrissa slipping along a straight edge and an analysis of frictional effects during whisking. *IEEE Trans. Haptics* 9 158–169. 10.1109/TOH.2016.2522432 26829805 PMC5753595

[B23] Huet L. A. Schroeder C. L. Hartmann M. J. Z. (2015). Tactile signals transmitted by the vibrissa during active whisking behavior. *J. Neurophysiol.* 113 3511–3518. 10.1152/jn.00011.2015 25867739 PMC4455487

[B24] Jones L. M. Lee S. Trageser J. C. Simons D. J. Keller A. (2004). Precise temporal responses in whisker trigeminal neurons. *J. Neurophysiol.* 92 665–668. 10.1152/jn.00031.2004 14999053 PMC2800049

[B25] Khatri V. Bermejo R. Brumberg J. C. Keller A. Zeigler H. P. (2009). Whisking in air: encoding of kinematics by trigeminal ganglion neurons in awake rats. *J. Neurophysiol.* 101 1836–1846. 10.1152/jn.90655.2008 19109457 PMC2695634

[B26] Kleinfeld D. Deschênes M. (2011). Neuronal basis for object location in the vibrissa scanning sensorimotor system. *Neuron* 72 455–468. 10.1016/j.neuron.2011.10.009 22078505 PMC3971931

[B27] Knutsen P. M. (2015). Whisking kinematics. *Scholarpedia* 10:7280 10.4249/scholarpedia.7280.

[B28] Knutsen P. Ahissar E. (2009). Orthogonal coding of object location. *Trends Neurosci.* 32 101–109. 10.1016/j.tins.2008.10.002 19070909

[B29] Knutsen P. M. Biess A. Ahissar E. (2008). Vibrissal kinematics in 3D: tight coupling of azimuth, elevation, and torsion across different whisking modes. *Neuron* 59 35–42. 10.1016/j.neuron.2008.05.013 18614027

[B30] Knutsen P. M. Pietr M. Ahissar E. (2006). Haptic object localization in the vibrissal system: behavior and performance. *J. Neurosci.* 26 8451–8464. 10.1523/JNEUROSCI.1516-06.2006 16914670 PMC6674338

[B31] Leiser S. C. Moxon K. A. (2007). Responses of trigeminal ganglion neurons during natural whisking behaviors in the awake rat. *Neuron* 53 117–133. 10.1016/j.neuron.2006.10.036 17196535

[B32] Lichtenstein S. H. Carvell G. E. Simons D. J. (1990). Responses of rat trigeminal ganglion neurons to movements of vibrissae in different directions. *Somatosen. Mot. Res.* 7 47–65. 10.3109/08990229009144697 2330787

[B33] Lottem E. Azouz R. (2011). A unifying framework underlying mechanotransduction in the somatosensory system. *J. Neurosci.* 31 8520–8532. 10.1523/JNEUROSCI.6695-10.2011 21653856 PMC6623321

[B34] Lottem E. Gugig E. Azouz R. (2015). Parallel coding schemes of whisker velocity in the rat’s somatosensory system. *J. Neurophysiol.* 113 1784–1799. 10.1152/jn.00485.2014 25552637

[B35] Mameli O. Caria M. A. Pellitteri R. Russo A. Saccone S. Stanzani S. (2016). Evidence for a trigeminal mesencephalic-hypoglossal nuclei loop involved in controlling vibrissae movements in the rat. *Exp. Brain Res.* 234 753–761. 10.1007/s00221-015-4503-6 26645304

[B36] Mameli O. Stanzani S. Mulliri G. Pellitteri R. Caria M. Russo A. (2010). Role of the trigeminal mesencephalic nucleus in rat whisker pad proprioception. *Behav. Brain Funct.* 6 1–12. 10.1186/1744-9081-6-69 21078134 PMC2993642

[B37] Mehta S. B. Whitmer D. Figueroa R. Williams B. A. Kleinfeld D. (2007). Active spatial perception in the vibrissa scanning sensorimotor system. *PLoS Biol.* 5:e15 10.1371/journal.pbio.0050015 PMC1769422 17227143

[B38] Mitchinson B. Grant R. A. Arkley K. Rankov V. Perkon I. Prescott T. J. (2011). Active vibrissal sensing in rodents and marsupials. *Philos. Trans. R. Soc. Lond. B Biol. Sci.* 366 3037–3048. 10.1098/rstb.2011.0156 21969685 PMC3172598

[B39] Moore J. D. Lindsay N. M. Deschenes M. Kleinfeld D. (2015). Vibrissa self-motion and touch are reliably encoded along the same somatosensory pathway from brainstem through thalamus. *PLoS Biol.* 13:e1002253 10.1371/journal.pbio.1002253 PMC4579082 26393890

[B40] O’Connor D. H. Clack N. G. Huber D. Komiyama T. Myers E. W. Svoboda K. (2010). Vibrissa-based object localization in head-fixed mice. *J. Neurosci.* 30 1947–1967. 10.1523/jneurosci.3762-09.2010 20130203 PMC6634009

[B41] Pammer L. O’connor D. H. Hires S. A. Clack N. G. Huber D. Myers E. W. (2013). The mechanical variables underlying object localization along the axis of the whisker. *J. Neurosci.* 33 6726–6741. 10.1523/JNEUROSCI.4316-12.2013 23595731 PMC3733083

[B42] Quist B. W. Faruqi R. A. Hartmann M. J. Z. (2011). Variation in Young’s modulus along the length of a rat vibrissa. *J. Biomech.* 44 2775–2781. 10.1016/j.jbiomech.2011.08.027 21993474

[B43] Quist B. W. Seghete V. Huet L. A. Murphey T. D. Hartmann M. J. Z. (2014). Modeling forces and moments at the base of a rat vibrissa during noncontact whisking and whisking against an object. *J. Neurosci.* 34 9828–9844. 10.1523/jneurosci.1707-12.2014 25057187 PMC4107402

[B44] Rutlin M. Ho C. Y. Abraira V. E. Cassidy C. Bai L. Woodbury C. J. (2014). The cellular and molecular basis of direction selectivity of a delta-LTMRs. *Cell* 159 1640–1651. 10.1016/j.cell.2014.11.038 25525881 PMC4297767

[B45] Simons D. J. (1978). Response properties of vibrissa units in rat S1 somatosensory neocortex. *J. Neurophysiol.* 41 798–820. 660231 10.1152/jn.1978.41.3.798

[B46] Simons D. J. (1985). Temporal and spatial integration in the rat S1 vibrissa cortex. *J. Neurophysiol.* 54 615–635. 4045540 10.1152/jn.1985.54.3.615

[B47] Solomon J. H. Hartmann M. J. Z. (2008). Artificial whiskers suitable for array implementation: accounting for lateral slip and surface friction. *IEEE Trans. Rob.* 24 1157–1167. 10.1109/tro.2008.2002562

[B48] Solomon J. H. Hartmann M. J. Z. (2010). Extracting object contours with the sweep of a robotic whisker using torque information. *Int. J. Rob. Res.* 29 1233–1245. 10.1177/0278364908104468

[B49] Solomon J. H. Hartmann M. J. Z. (2011). Radial distance determination in the rat vibrissal system and the effects of Weber’s law. *Philos. Trans. R. Soc. Lond. B Biol. Sci.* 366 3049–3057. 10.1098/rstb.2011.0166 21969686 PMC3172605

[B50] Szwed M. Bagdasarian K. Ahissar E. (2003). Encoding of vibrissal active touch. *Neuron* 40 621–630. 10.1016/S0896-6273(03)00671-8 14642284

[B51] Szwed M. Bagdasarian K. Blumenfeld B. Barak O. Derdikman D. Ahissar E. (2006). Responses of trigeminal ganglion neurons to the radial distance of contact during active vibrissal touch. *J. Neurophysiol.* 95 791–802. 10.1152/jn.00571.2005 16207785

[B52] Towal R. B. Hartmann M. J. (2006). Right-left asymmetries in the whisking behavior of rats anticipate head movements. *J. Neurosci.* 26 8838–8846. 10.1523/jneurosci.0581-06.2006 16928873 PMC6674387

[B53] Towal R. B. Hartmann M. J. (2008). Variability in velocity profiles during free-air whisking behavior of unrestrained rats. *J. Neurophysiol.* 100 740–752. 10.1152/jn.01295.2007 18436634

[B54] Towal R. B. Quist B. W. Gopal V. Solomon J. H. Hartmann M. J. Z. (2011). The morphology of the rat vibrissal array: a model for quantifying spatiotemporal patterns of whisker-object contact. *PLoS Comp. Biol.* 7:e1001120 10.1371/journal.pcbi.1001120 PMC3072363 21490724

[B55] Wallach A. Bagdasarian K. Ahissar E. (2016). On-going computation of whisking phase by mechanoreceptors. *Nat. Neurosci.* 19 487–493. 10.1038/nn.4221 26780508

[B56] Whiteley S. J. Knutsen P. M. Matthews D. W. Kleinfeld D. (2015). Deflection of a vibrissa leads to a gradient of strain across mechanoreceptors in a mystacial follicle. *J. Neurophysiol.* 114 138–145. 10.1152/jn.00179.2015 25855692 PMC4507969

[B57] Williams C. M. Kramer E. M. (2010). The advantages of a tapered whisker. *PLoS ONE* 5:e8806 10.1371/journal.pone.0008806 PMC2808387 20098714

[B58] Zucker E. Welker W. I. (1969). Coding of somatic sensory input by vibrissae neurons in rats trigeminal ganglion. *Brain Res.* 12 138–156. 10.1016/0006-8993(69)90061-4 5802473

